# A maltose-regulated large genomic region is activated by the transcriptional regulator MalT in *Actinoplanes* sp. SE50/110

**DOI:** 10.1007/s00253-020-10923-2

**Published:** 2020-09-28

**Authors:** Julian Droste, Martin Kulisch, Timo Wolf, Lena Schaffert, Susanne Schneiker-Bekel, Alfred Pühler, Jörn Kalinowski

**Affiliations:** 1 Microbial Genomics and Biotechnology, Center for Biotechnology, Bielefeld University, Universitätsstraße 27, 33615 Bielefeld, Germany; 2 Senior Research Group in Genome Research of Industrial Microorganisms, Center for Biotechnology, Bielefeld University, Universitätsstraße 27, 33615 Bielefeld, Germany

**Keywords:** *Actinoplanes*, Transcriptomic, Transcriptional regulation, MalT

## Abstract

**Abstract:**

*Actinoplanes* sp. SE50/110 is the industrially relevant producer of acarbose, which is used in the treatment of diabetes mellitus. Recent studies elucidated the expression dynamics in *Actinoplanes* sp. SE50/110 during growth. From these data, we obtained a large genomic region (*ACSP50_3900* to *ACSP50_3950*) containing 51 genes, of which 39 are transcribed in the same manner. These co-regulated genes were found to be stronger transcribed on maltose compared with glucose as a carbon source. The transcriptional regulator MalT was identified as an activator of this maltose-regulated large genomic region (MRLGR). Since most of the genes are poorly annotated, the function of this region is farther unclear. However, comprehensive BLAST analyses indicate similarities to enzymes involved in amino acid metabolism. We determined a conserved binding motif of MalT overlapping the -35 promoter region of 17 transcription start sites inside the MRLGR. The corresponding sequence motif 5′-TCATCC-5nt-GGATGA-3′ displays high similarities to reported MalT binding sites in *Escherichia coli* and *Klebsiella pneumoniae*, in which MalT is the activator of *mal* genes. A *malT* deletion and an overexpression mutant were constructed. Differential transcriptome analyses revealed an activating effect of MalT on 40 of the 51 genes. Surprisingly, no gene of the maltose metabolism is affected. In contrast to many other bacteria, MalT is not the activator of *mal* genes in *Actinoplanes* sp. SE50/110. Finally, the MRLGR was found partly in other closely related bacteria of the family Micromonosporaceae. Even the conserved MalT binding site was found upstream of several genes inside of the corresponding regions.

****Key points**:**

• *MalT is the maltose-dependent activator of a large genomic region in ACSP50_WT.*

• *The consensus binding motif is similar to MalT binding sites in other bacteria.*

• *MalT is not the regulator of genes involved in maltose metabolism in ACSP50_WT.*

**Electronic supplementary material:**

The online version of this article (10.1007/s00253-020-10923-2) contains supplementary material, which is available to authorized users.

## Introduction

*Actinoplanes* sp. SE50/110 is the natural producer of the pseudotetrasaccharide acarbose (acarviosyl-1,4-maltose), which functions as an α-glucosidase inhibitor and is used in the treatment of diabetes mellitus (Truscheit et al. 1981). It is a Gram-positive, aerobic bacterium, which grows in branched hyphae and can form sporangia and motile spores (Vobis et al. 2015). The genome of *Actinoplanes* sp. SE50/100 has a high G+C content of 71.32%, which was first sequenced by Schwientek et al. (2012). Today, a refined high-quality genome of *Actinoplanes* sp. SE50/110 is available (Wolf et al. 2017b).

*Actinoplanes* spp. are known for their potential to produce a variety of secondary metabolites and antibiotics, like actaplanin (Debono et al. 1984), friulimicins (Aretz et al. 2000), moenomycin (Horbal et al. 2016), ramoplanin (Ciabatti et al. 1989), and teicoplanin (Bardone et al. 1978). Mining the genomes of actinomycetes, gene clusters for the production of several industrially relevant products could be identified. Also, the genome of *Actinoplanes* sp. SE50/110 harbors about 20 gene clusters, which are potentially responsible for the biosynthesis of secondary metabolites (Wolf et al. 2017b).

*Actinoplanes* sp. SE50 strains are industrially relevant producer of acarbose (Wehmeier and Piepersberg 2004). Therefore, understanding of the metabolism and the regulatory processes of this bacterium is an important step to optimize acarbose-producing conditions and to identify potential targets for metabolic engineering in order to increase acarbose productivity in the future.

Recent studies analyzed expression dynamics of all genes and operons during growth (Droste et al. 2020). Many co-regulated genes were identified by hierarchical cluster analyses, such as the *acb* gene cluster responsible for acarbose biosynthesis. A total of 71 genes were found to be transcribed coordinately, showing an increasing transcript amount during growth (Cluster 36, Droste et al. 2020). Interestingly, 41 genes were found to be located in close proximity in a region comprised of 51 genes (*ACSP50_3900* to *ACSP50_3950*). Differential transcriptome analyses revealed an increased transcription of this genomic region on maltose compared with glucose as a carbon source (Supplemental Fig. S1 and Supplemental Table S1).

In this study, we analyzed this maltose-regulated large genomic region (MRLGR) and its transcriptional regulation. A conserved sequence motif analysis was applied to prove co-regulation of these genes. Interestingly, only two transcriptional regulator genes (*ACSP50_3915* and *ACSP50_3917*) were found inside the MRLGR. We investigated the effects of the transcriptional regulator MalT (*ACSP50_3915*) on the surrounding genes by deletion and overexpression. Furthermore, we analyzed the potential function of the corresponding gene products.

## Materials and methods

### Strains, media, and cultivation conditions

All cloning procedures were carried out with *Escherichia coli* DH5αMCR (Grant et al. 1990). For the conjugational transfer of plasmids into *Actinoplanes* sp. SE50/110 (ATCC21044), the strain *E. coli* ET12567 (pUZ8002) (Kieser et al. 2004) was used as a conjugation host to generate the mutated strains of *Actinoplanes* in this study.

For *malT* (*ACSP50_3915*), the gene deletion CRISPR/Cas9 technique based on the plasmid pCRISPomyces-2 was used as described by Wolf et al. (2016). Spacer for the generation of the guide RNA (gRNA) and primer for amplification and cloning of up- and downstream flanking sequences are listed in Supplemental Table S2. Cloning procedures were carried out according to Cobb et al. (2014) and Wolf et al. (2016). The deletion plasmid was transferred into *Actinoplanes* sp. SE50/110 by conjugation as described before (Gren et al. 2016). The successful deletion of *malT* was proven by PCR and Sanger sequencing with primers listed in Supplemental Table S2. Gene deletion resulted in the strain *Actinoplanes* sp. SE50/110 Δ*malT* (referred to as ACSP50_Δ*malT* in this study).

A *malT* overexpression plasmid was constructed based on the integrative vector pSET152 (Gren et al. 2016) using the strong promoter P_*gapDH*_ from *Eggerthella lenta* (Schaffert et al. 2019a), resulting in the strain *Actinoplanes* sp. SE50/110 pSET152::P_*gapDH*_::*malT* (referred to as ACSP50_OE*malT* in this study). The strain *Actinoplanes* sp. SE50/110 pSET152 (referred to as ACSP50_pSET in this study) containing the plasmid pSET152 was used as an empty vector control.

The *Actinoplanes* sp. SE50/110 wild type strain (referred to as ACSP50_WT in this study), and the mutants (ACSP50_pSET, ACSP50_Δ*malT*, ACSP50_OE*malT*) derived from this strain were grown on soy flour medium (SFM; 20-g L^−1^ soy, 20-g L^−1^ mannitol, 20-g L^−1^ agar, pH 8.0, tap water) agar plates and in NBS (11 g L^−1^ glucose × 1 H_2_O, 4 g L^−1^ peptone, 4 g L^−1^ yeast extract, 1 g L^−1^ MgSO_4_ × 7 H_2_O, 2 g L^−1^ KH_2_PO_4_, 4 g L^−1^ K_2_HPO_4_) complex medium. For shake flask cultivations, minimal medium supplemented with maltose or glucose as a carbon source was used as described elsewhere (Wendler et al. 2015).

Shake flask cultivations were carried out in five biological replicates in 250-mL Corning® Erlenmeyer baffled cell culture flasks. Therefore, 50 mL of minimal medium was inoculated with spore suspension obtained from bacterial strains grown on SFM agar plates for 6 to 7 days at 28 °C and harvested with 1 mL ddH_2_O. Cell growth was examined by the determination of cell dry weight. For RNA isolation and subsequent transcriptome analyses, 1 mL cell suspension was centrifuged for 15 s at maximum speed and immediately frozen in liquid nitrogen. Cell pellets were stored at − 80 °C until RNA isolation (Wolf et al. 2017a).

### RNA isolation and transcriptome analysis

#### RNA isolation

For the transcriptome analysis, RNA was isolated using a Macherey-Nagel NucleoSpin® RNA Plus kit in combination with Macherey-Nagel rDNase Set (Macherey-Nagel, Düren, Germany). Therefore, cell pellets were resuspended in 500 μL LBP buffer (NucleoSpin® RNA Plus kit, Macherey-Nagel) and transferred into 2-mL lysing matrix tubes (0.1-mm spherical silica beads, MP Biomedicals, Santa Ana, CA, USA). Cell disruption was carried out in a homogenizer (FastPrep FP120, Thermo Fisher Scientific, Waltham, MA, USA) two times for 30 s at speed setting 6.5 and 1 min on ice in between. Following this, cell debris were centrifuged for 2 min at maximum speed at 4 °C. The supernatant was used for RNA isolation according to the manufacturer’s protocol. To verify the complete removal of residual DNA in the samples, PCR with primers binding to genomic *Actinoplanes* sp. SE50/110 DNA was performed. Quality and quantity of the RNA were analyzed with a NanoDrop 1000 spectrometer (Peqlab, Erlangen, Germany) and an Agilent RNA 6000 Pico kit run on an Agilent Bioanalyzer 2100 (Agilent Technologies, Santa Clara, CA, USA).

#### Whole-genome oligonucleotide microarray

Custom whole-genome oligonucleotide microarrays representing nearly all coding sequences of *Actinoplanes* sp. SE50/110 were used as described previously (Wolf et al. 2017a). Summarized, Agilent custom microarrays in the 4x44K format were used with a Two-Color Microarray-Based Prokaryote Analysis FairPlay III Labeling kit (version 1.4, Agilent Technologies, Santa Clara, CA, USA). After feature extraction using the manufacturer’s software package, data analysis was performed with the software EMMA2 (Dondrup et al. 2009). The data was normalized (LOWESS) and a *t* test (one-sample, Holm) was applied. A *p* value of 0.05 was used as a cutoff for significance, and the log2 (ratio) cutoffs for a false discovery rate of 0.01 were experimentally determined as 1.1 and − 1.1 (Wolf et al. 2017a).

#### Reverse transcription quantitative PCR

Reverse transcription quantitative PCR (RT-qPCR) was carried out using a Bioline SensiFast SYBR No-Rox One-Step Kit (Bioline, London, UK) in 96-well LightCycler plates (Sarstedt, Nümbrecht, Germany) and measured in a Roche LightCycler 96 System (Roche, Mannheim, Germany). Samples were prepared according to the protocol described by Wolf et al. (2017a). The relative transcript amount was calculated as 2^-ΔCq^, whereas ΔCq was determined as difference of the mean Cq in the mutated strain compared with the respective control strain. Primers used for the RT-qPCR are listed in Supplemental Table S2.

## Results

### Forty-one genes of the maltose-regulated large genomic region are co-regulated in *Actinoplanes* sp. SE50/110

**Fig. 1:**
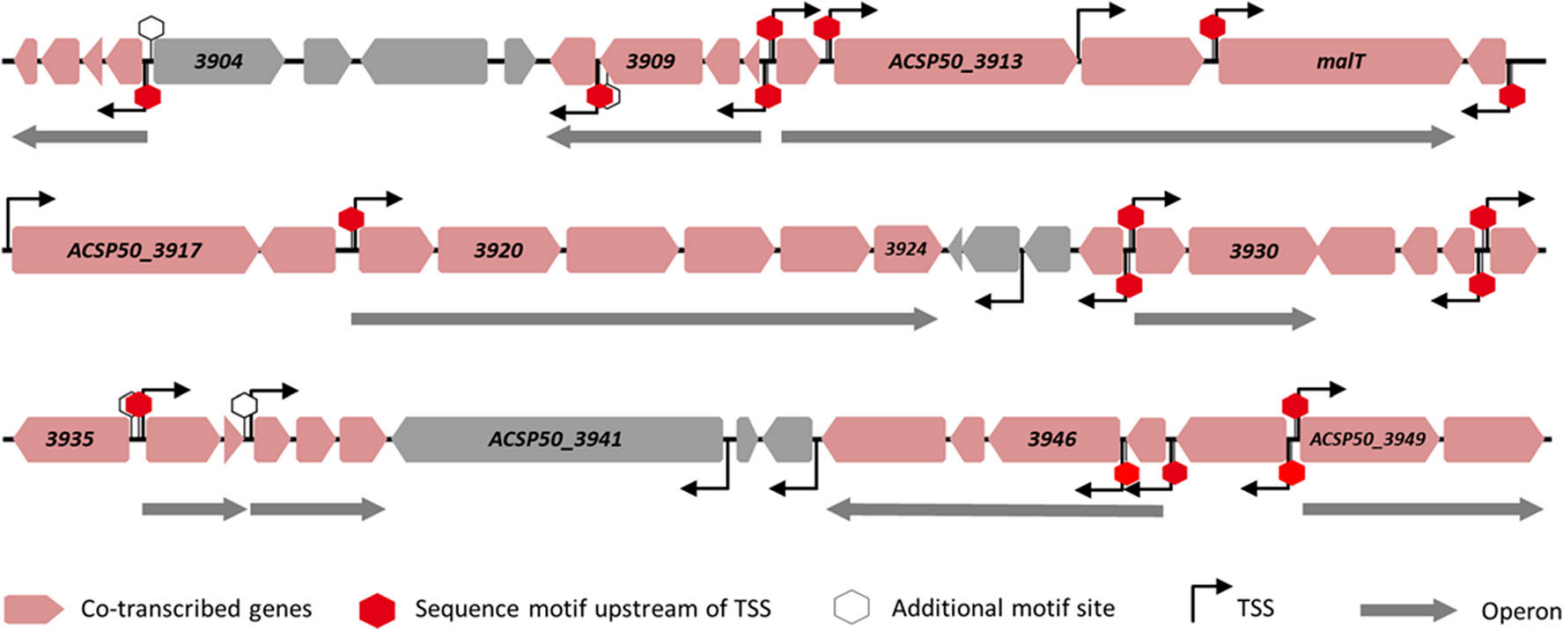
Maltose-regulated large genomic region (MRLGR) ranging from ACSP50_3900 to ACSP50_3950 in *Actinoplanes* sp. SE50/110. Genes found to be co-regulated during growth are marked in light red. Data are obtained from Droste et al. (2020). Transcription start sites (TSS) and operon structure are indicated by black and gray arrows respectively. Additionally, locations of the sequence motif shown in Fig. 2 are marked with hexagons. If the sequence motif was found upstream of a TSS, the position is marked with a red hexagon, whereas additional locations of the motif are visualized in white, black-edged hexagons. The annotations of these genes are listed in Supplemental Table S3

In recent studies, several co-expressed genes were identified by transcriptome and proteome analyses during growth of *Actinoplanes* sp. SE50/110 (Droste et al. 2020). These genes might belong to the same regulons. A large genomic region of 51 genes (ACSP50_3900 to ACSP50_3950) was found to be transcribed coordinately during growth. For 41 of the 51 genes, a highly similar continuously increasing transcript amount over the course of the cultivation was determined by hierarchical cluster analysis (Droste et al. 2020). Interestingly, we also identified this genomic region by comparative transcriptome analysis of *Actinoplanes* sp. SE50/110 wild type strain grown on maltose compared with glucose minimal medium (Supplemental Fig. S1 and Supplemental Table S1). The aim of this experiment was to identify genes with an increased transcript amount on maltose as a carbon source compared with glucose. Cells were cultivated in minimal medium, and samples for transcriptome analysis were taken after 72 h (Supplemental Fig. S1). By analyzing the top scorer of this experiment (genes which are highly transcribed on maltose compared with glucose), it could be shown that the genes of the region ACSP50_3900 to ACSP50_3950 are among the genes with the highest *M* values. This way, it was shown that these genes are stronger transcribed on maltose compared with glucose. The similar transcription pattern, the close proximity of these genes, and the maltose-dependent expression indicate a maltose-dependent co-regulation of this genomic region during growth. Therefore, this genomic region (Fig. 1) was named maltose-regulated large genomic region.

Analyzing the annotation of these genes, no gene products involved in the maltose metabolism of *Actinoplanes* sp. SE50/110 could be identified (Schaffert et al. 2019b). Twenty-four of the 51 genes have no annotated function (“hypothetical protein,” “uncharacterized protein”) according to the NCBI database (GenBank: LT827010). Interestingly, 10 genes were annotated as membrane or transport related. Further 17 genes were annotated as peptidases, transferases, glucosidases, and other enzymes. Finally, two transcriptional regulator genes could be found inside the MRLGR (ACSP50_3915 and ACSP50_3917). Interestingly, both are annotated as LuxR family (MalT-like) transcriptional regulators.

### A conserved palindromic sequence motif was identified in the promoter region of 17 genes of the MRLGR in *Actinoplanes* sp. SE50/110

**Fig. 2:**
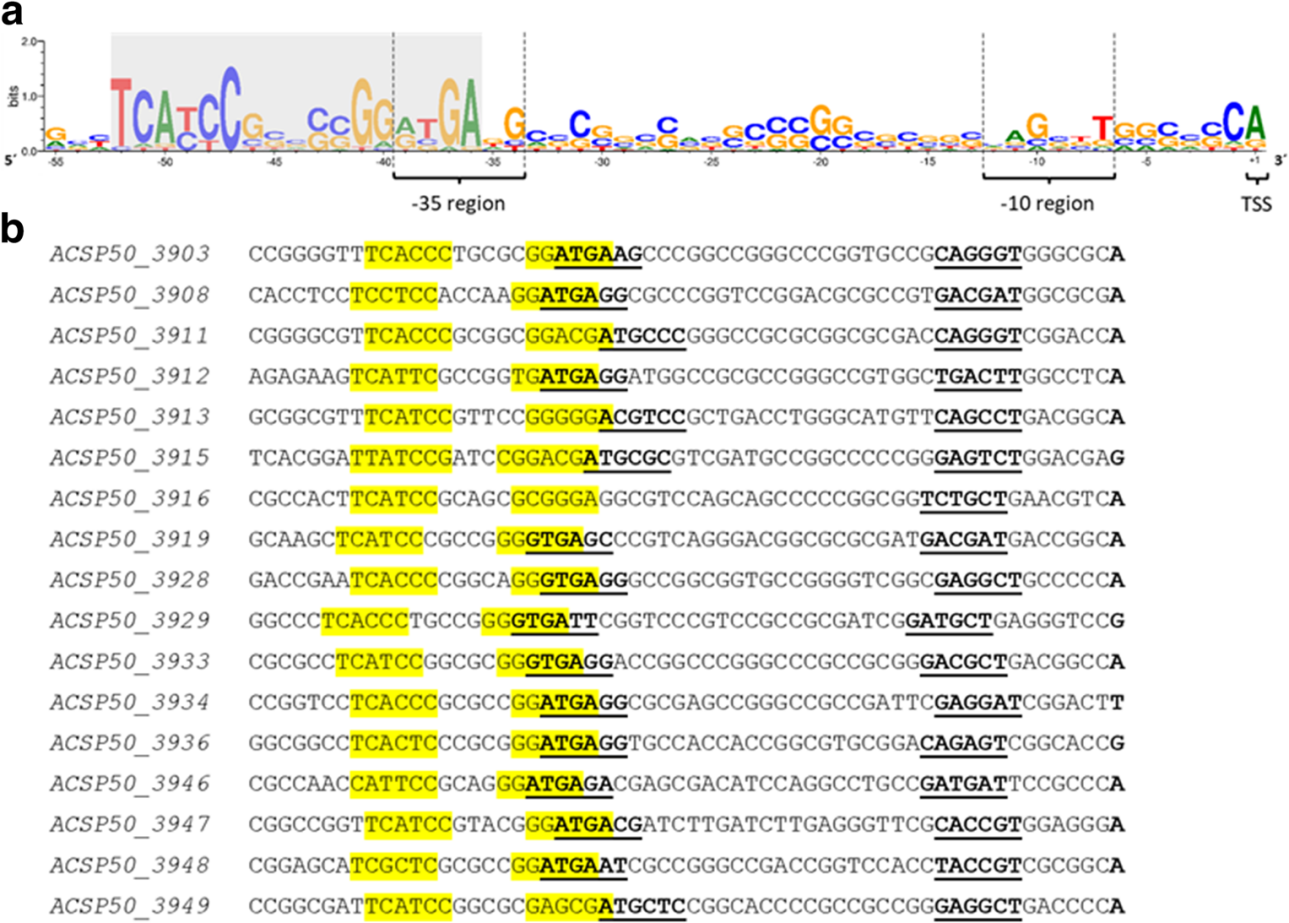
TSS upstream sequences of genes co-regulated in the MRLGR (ACSP50_3900 to ACSP50_3950) in *Actinoplanes* sp. SE50/110 with an assigned TSS. The TSS are assigned according to Wolf et al. (2017b) and Droste et al. (2020) (submitted to BMC Genomics). **a** Consensus sequence of the promoter region of 17 genes of the MRLGR in *Actinoplanes* sp. SE50/110. The promoter motifs (-10 and -35 region) are marked with dashed lines. A conserved palindromic sequence motif overlapping the -35 region is highlighted in gray. **b** TSS upstream sequences used for consensus sequence shown in **a**. The corresponding TSS and promoter elements are shown in bold letters. The -10 and -35 regions are underlined. The palindromic sequence motif site is marked in yellow

The strict co-regulation of genes in bacteria is likely to be caused by a transcriptional regulator. Therefore, binding sites for transcription factors or alternative sigma factors might be conserved upstream of the transcription start sites (TSS) of these genes. Since many genes are organized in operons, a TSS was not identified upstream of every gene (Droste et al. 2020). Therefore, the transcription is initiated at the same sequence position for several genes. For 23 genes of this MRLGR, at least one TSS could be identified using the dataset of Droste et al. (2020). The tool MEME (Bailey et al. 2009) was used to identify motifs within the sequences 71 bp upstream of the TSS (− 70 to + 1) of these genes. A palindromic hexanucleotide sequence (5′-TCATCC-5 nt-GGATGA-3′) was identified in 17 sequences with an *e* value of 5.4 × 10^−29^ (Fig. 2). The distance to the upstream TSS was determined as 34.4 ± 1.0 bases from the 3′ end of the conserved motif and therefore overlaps with the -35 region of the corresponding promoters. This type of motif hints toward a characteristic binding site for transcription factors (Rhodes et al. 1996; Huffman and Brennan 2002). The identification of the transcriptional regulator responsible for maltose-dependent regulation of the MRLGR in *Actinoplanes* sp. SE50/110 is discussed in the chapter after next.

### Functional analysis of the proteins encoded by the MRLGR in *Actinoplanes* sp. SE50/110

It could be shown that at least 41 of 51 genes of the MRLGR are strictly co-regulated dependent on the presence of maltose. However, the function of most of these genes belonging to this regulon is unclear, since they were poorly annotated by the automated annotation software pipeline Prokka, version 1.11 (Seemann 2014). The annotated functions of these genes according to the NCBI database (GenBank: LT827010) are listed in Supplemental Table S3. Therefore, we used the tools KEGG mapper (Kanehisa and Sato 2020; Kanehisa et al. 2016a) and GhostKOALA (Kanehisa et al. 2016b) in this work to further analyze the potential function of the genes of the MRLGR in *Actinoplanes* sp. SE50/110 (Supplemental Table S3). Interestingly, no common pathway or metabolism could be identified containing a significant number of proteins encoded by the MRLGR, although co-regulation of these genes was observed. However, the strongest commonality was found for 10 proteins, which contain domains that are similar to enzymes of the amino acid metabolism (Table 1). Therefore, we assume that parts of the MRLGR products are involved in the amino acid metabolism, such as arginine biosynthesis. For most of these proteins, at least one homologous gene/protein was identified in the genome of *Actinoplanes* sp. SE50/110 (Table 1).

**Table 1:** Annotated function of 10 genes inside the MRLGR. The putative metabolic pathway and homologous genes in the genome of ACSP50_WT were listed

Locus tag	Annotated function (Wolf et al. 2017b)	Metabolic pathway	Homologous genes^1^ in ACSP50_WT
*ACSP50_3919*	Class II glutamine amidotransferase	Amino acid metabolism	*ACSP50_6409*
*ACSP50_3920*	Amino acid permease	Amino acid metabolism	*ACSP50_2706*; *ACSP50_3876*
*ACSP50_3921*	Arginine deiminase	Arginine biosynthesis	*ACSP50_8316*
*ACSP50_3922*	Ornithine carbamoyltransferase	Arginine biosynthesis	*ACSP50_4060*
*ACSP50_3923*	Carbamate kinase	Arginine biosynthesis	*ACSP50_6398*
*ACSP50_3924*	Cyclic nucleotide–binding protein (phosphodiesterase)	Put. serine/threonine biosynthesis	
*ACSP50_3944*	Beta-Ala-His dipeptidase	Amino acid metabolism	*ACSP50_1214*
*ACSP50_3946*	Amino acid permease	Amino acid metabolism	
*ACSP50_3948*	Threonine/serine exporter family protein	Serine/threonine biosynthesis	
*ACSP50_3950*	Aminopeptidase P family protein	Amino acid metabolism	*ACSP50_1832*

^1^Revealed by BLASTP analysis, *e* value < 7e^−14^

The enzymes ACSP50_3921, ACSP50_3922, and ACSP50_3923 are potentially involved in the arginine biosynthesis. By in silico analysis of the respective enzymatic reactions, a flux toward citrulline from ornithine and arginine could be observed.

A comprehensive BLAST analysis by the algorithms BLASTP and tBLASTn (Altschul et al. 1997) of the genomic region *ACSP50_3900* to *ACSP50_3950* was performed using respective protein sequences as input data. The full list of BLAST analysis can be found in Supplemental Table S3.

**Fig. 3:**
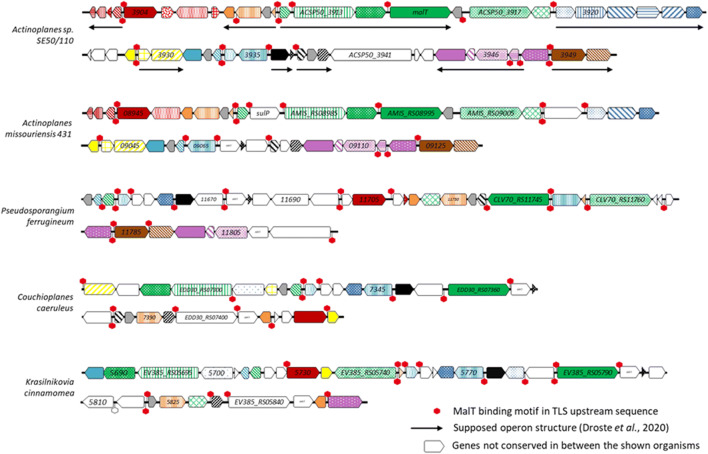
Comparison of the MRLGR with similar genomic regions of *Actinoplanes missouriensis*, *Pseudosporangium ferrugineum*, *Couchioplanes caeruleus*, and *Krasilnikovia cinnamomea* containing homologous gene products identified by BLAST analysis. Genes of homologous proteins are marked in the same color code. The positions of the conserved sequence motif (5′-TCATCC-5 bp-GGATGA-3′) in all strains are marked with red (upstream of ORF) and white (additional sites) hexagons. A detailed list of all shown genes and their annotated function is given in Supplemental Table S4

The results of the BLAST analyses revealed high similarities of the proteins to three different organisms: *Pseudosporangium ferrugineum*, *Couchioplanes caeruleus*, and *Krasilnikovia cinnamomea* (Fig. 3). The genomes of these three bacteria were searched for a similar genomic region compared with the MRLGR of *Actinoplanes* sp. SE50/110. In addition, the corresponding genomic region of the close relative *Actinoplanes missouriensis* was analyzed for comparison. For all strains, at least 23 genes homologous to genes from *ACSP50_3900* to *ACSP50_3950* were found to be located in close proximity to each other. However, not all genes were found in the same order and direction. Genes, which seem to be organized in operons in *Actinoplanes* sp. SE50/110, are rearranged in the other strains (Fig. 3). Even between the two *Actinoplanes* spp., clear differences were identified regarding this genomic region.

For the regulator gene *malT* (ACSP50_3915), a homologous gene could be identified in all analyzed genomes (Fig. 3), whereas ACSP50_3917 homologs were only identified in three of four species. By this, ACSP50_3915 is more conserved compared with ACSP50_3917. We assume that ACSP50_3915 is the key regulator of the surrounding genes.

Interestingly, several genes which were not found to be co-regulated in *Actinoplanes* sp. SE50/110 (Fig. 1), like *ACSP50_3904* to *ACSP50_3907*, *ACSP50_3925* to *ACSP50_3927*, or *ACSP50_3941* to *ACSP50_3943*, are not conserved between the analyzed strains (Supplemental Table S4), except for *ACSP50_3904*. No homologous genes could be identified in the compared strains, not even in the close relative *A. missouriensis*. Furthermore, the operon *ACSP50_3920* to *ACSP50_3924* is lacking in the four analyzed bacterial genomes, except for *ACSP50_3924* encoding a cyclic nucleotide–binding protein, which was identified in all strains. Additionally, *A. missouriensis* contains an *ACSP50_3921* homolog coding for an arginine deiminase. Strikingly, genes encoding a polyphosphate kinase (*ppk2*) were found in one or even two copies in the corresponding genomic regions of the analyzed bacteria but lack in the MRLGR of *Actinoplanes* sp. SE50/110.

Finally, the palindromic sequence motif identified in the -35 region of the MRLGR genes could also be identified upstream of several open reading frames (ORFs) in the analyzed genomic regions of *A. missouriensis*, *P. ferrugineum*, *C. caeruleus*, and *K. cinnamomea* (Fig. 3). This confirms the close relation of these genomic regions.

On the one hand, several genes of this region seem to be highly conserved as well as the identified palindromic sequence motif upstream of the ORFs. On the other hand, the arrangement and order of these genes are highly diverse comparing different bacterial strains. Therefore, it can be assumed that this genomic region was passed on by horizontal gene transfer in several related species of the family Micromonosporaceae. We assume that the gene products of this region are important, but not all are essential for each respective strain. Especially for growth on glucose, most of the genes seem to be low or not transcribed at all in *Actinoplanes* sp. SE50/110.

### The transcriptional regulator MalT (ACSP50_3915) is the activator of the MRLGR in *Actinoplanes* sp. SE50/110

The observations above lead to the conclusion that the genes of the MRLGR are strictly regulated by one common transcriptional regulator. Since only two transcriptional regulator genes (*ACSP50_3915* and *ACSP50_3917*) could be identified in the MRLGR, it was assumed that at least one of these regulators is responsible for regulation of the MRLGR.

However, only *ACSP50_3915* (*M* value of 2.23) but not *ACSP50_3917* (*M* value of 0.24) was found to be transcriptionally “upregulated” on maltose compared with glucose in our transcriptome analysis (Supplemental Fig. S1). In addition, a higher protein similarity was found for ACSP50_3915 (42% similarity) to MalT in *E. coli* (Supplemental Fig. S2). Therefore, it was assumed that MalT (ACSP50_3915) might be the transcriptional regulator of the MRLGR.

In order to prove this regulatory function, the corresponding gene *ACSP50_3915* was deleted using CRISPR/Cas9 (Wolf et al. 2016), resulting in an *Actinoplanes* sp. SE50/110 Δ*malT* deletion mutant (ACSP50_Δ*malT*). In addition, *malT* was overexpressed in *Actinoplanes* sp. SE50/110 using the strong promoter P_*gapDH*_ from *Eggerthella lenta* (Schaffert et al. 2019a) combined with the integrative vector pSET152 (Gren et al. 2016), resulting in the *malT* overexpression strain ACSP50_OE*malT*.

**Fig. 4:**
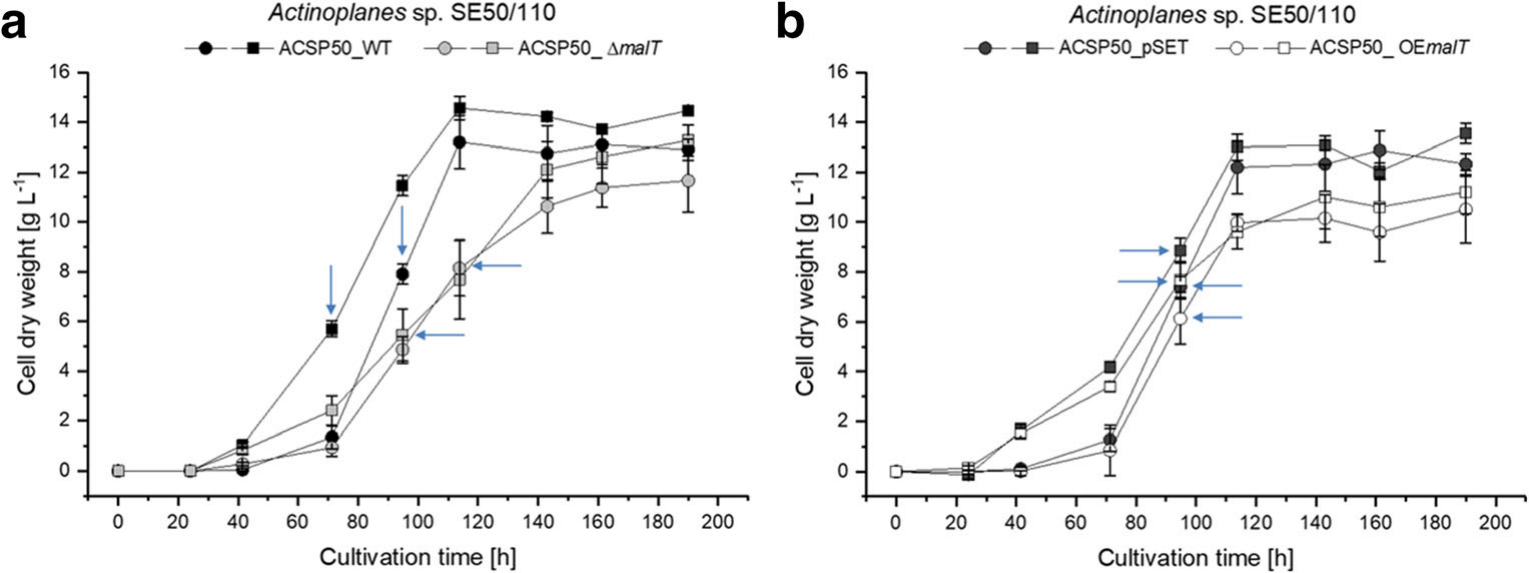
Growth of ACSP50_WT (black), ACSP50_Δ*malT* (gray), and ACSP50_OE*malT* (white). Cell dry weight for cells grown in minimal medium inoculated with spores supplemented with glucose (circles) and maltose (squares) as a carbon source. The means and standard deviations of five biological and two technical replicates are shown. Sampling points for transcriptome analysis are indicated with blue arrows

Both constructed mutant strains ACSP50_Δ*malT* and ACSP50_OE*malT* were cultivated in comparison with the *Actinoplanes* sp. SE50/110 wild type strain (ACSP50_WT) and an empty vector control strain (ACSP50_pSET) in a shake flask cultivation in minimal medium supplemented with maltose and glucose as a carbon source (Fig. 4). It could be shown that the regulator deletion mutant ACSP50_Δ*malT* grows slightly slower under both conditions (maltose and glucose) compared with the wild type (Fig. 4a). The *malT* overexpression strain (ACSP50_OE*malT*) shows significantly reduced growth on both glucose and maltose as a carbon source compared with an empty vector control (Fig. 4b).

Samples for transcriptome analysis were taken in the middle of the growth phase (after 96 h) of all strains on maltose and glucose each (Fig. 4), except for ACSP50_WT grown on maltose (transcriptome samples after 72 h) and ACSP50_Δ*malT* on glucose (transcriptome samples after 110 h) (Fig. 4a). The RNA was isolated and pooled from three biological replicates. Transcriptome analysis was carried out using whole-genome microarrays as described elsewhere (Wolf et al. 2017a).

**Fig. 5:**
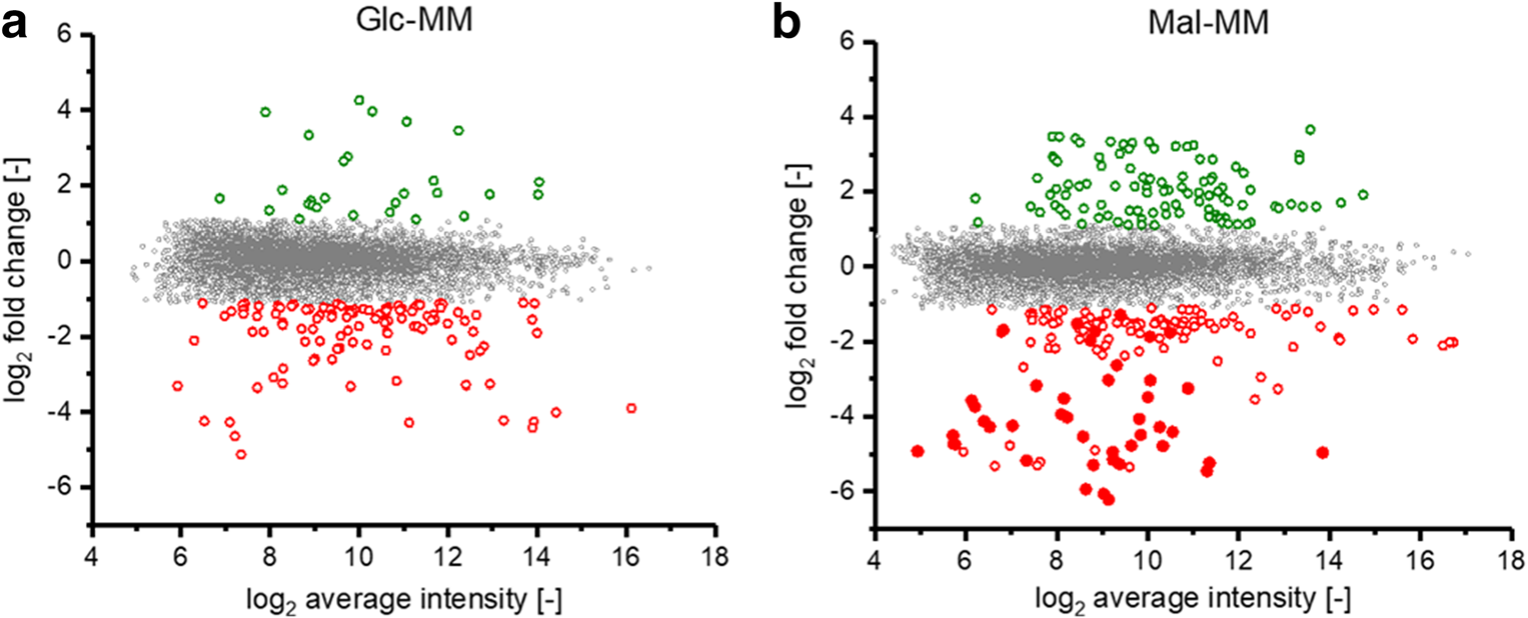
Differential transcriptional analysis of ACSP50_Δ*malT* compared with ACSP50_WT. **a** Ratio/intensity plot from whole-genome microarrays of the Δ*malT* mutant compared with the wild type grown in glucose minimal medium (Glc-MM). **b** Ratio/intensity plot from whole-genome microarrays of the Δ*malT* mutant compared with the wild type grown in maltose minimal medium (Mal-MM). Green and red dots represent genes with significantly different transcript levels in the Δ*malT* strain (*M* value > 1.1 or < − 1.1 respectively; *p*_adj_ value > 0.05). Filled dots show genes of the MRLGR

In total, 141 genes were found to be significantly differentially transcribed on glucose, of which 28 genes show an increased and 113 genes a decreased transcript amount in ACSP50_Δ*malT* compared with the wild type strain (Fig. 5). On maltose as a carbon source, 247 genes with significant differential transcription were identified (101 increased and 146 decreased transcript amount). Strikingly, only 69 (11 increased and 58 decreased) differentially transcribed genes were found under both conditions (Supplemental Table S5). In addition to three genes annotated as hypothetical or uncharacterized proteins, two genes with a membrane-associated gene product (*ACSP50_0484*, *ACSP50_2520*), two RNA polymerase sigma-24 subunits (*ACSP50_3334*, *ACSP50_3840*), a polyhydroxyalkanoate depolymerase (*ACSP50_3332*), a NAD-dependent deacetylase (*ACSP50_4603*), an epimerase (*ACSP50_4604*), and a serine hydrolase (*ACSP50_8214*) were found to be “transcriptionally upregulated” under both conditions in ACSP50_Δ*malT*. Among the 58 genes, which show a significantly decreased transcript amount under both conditions, 18 genes with no annotated function, two glutathione-dependent formaldehyde dehydrogenases (*ACSP50_1264*, *ACSP50_4381*), 8 genes annotated as membrane proteins or transporters, an anti-sigma factor (*ACSP50_0205*), a glycosyl transferase (*ACSP50_2948*), a transglycosylase (*ACSP50_1322*), and a trehalose synthase (*ACSP50_7524*) were identified. All results of the differential transcriptome analysis can be found in Supplemental Table S5.

Interestingly, the transcription of the MRLGR was strongly influenced by the deletion of the *malT* gene on maltose minimal medium but not on glucose (Fig. 5). This can be explained by the fact that the genes are not transcribed on glucose at all in the wild type, which is why no effect on the respective genes is visible on glucose (Supplemental Fig. S1 and Supplemental Table S1). A total of 32 of all 51 genes of the MRLGR are significantly less transcribed (*p*_adj_ < 0.05; *M* value < 1.1) in ACSP50_Δ*malT* on maltose as a carbon source, whereas 41 were previously described to be co-regulated in *Actinoplanes* sp. SE50/110 (Fig. 1, Droste et al. 2020). However, all genes of the MRLGR, which were identified to be less transcribed in ACSP50_Δ*malT*, were also previously described as co-regulated, except for *ACSP50_3907*. Additionally, 23 of the 31 genes, which were both described as co-regulated as well as significantly downregulated in ACSP50_Δ*malT*, were found to be significantly upregulated on maltose compared with glucose (Supplemental Table S6). These matches indicate that MalT is the maltose-dependent transcriptional activator of these genes. Strikingly, no genes of the maltose metabolism were found to be significantly different transcribed in ACSP50_Δ*malT* compared with ACSP50_WT. The maltase AmlE (*ACSP50_2474*), which was described to be essential for maltose degradation (Schaffert et al. 2019a, 2019b), shows an *M* value of − 0.393 (*p*_adj_ value > 0.5), or the operon *malEFG*, which was described to encode the maltose import system of *Actinoplanes* sp. SE50/110 (Wendler et al. 2016), exhibits *M* values of − 0.162 to 0.213 (*p*_adj_ values > 0.5) in the mutant strain compared with the wild type both grown on maltose minimal medium (Supplemental Table S5).

**Fig. 6:**
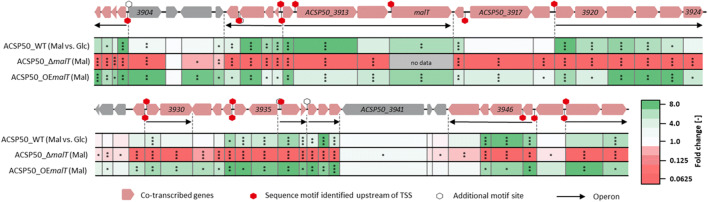
Differential transcriptional analysis of the MRLGR of ACSP50_WT under different expression levels of the transcriptional activator MalT (ACSP50_3915) and on different carbon sources. The values for ACSP50_WT on maltose (Mal) compared with glucose (Glc) (green color indicates increased transcription on maltose) as well as the deletion mutant ACSP50_Δ*malT* and the *malT* overexpression strain ACSP50_OE*malT* on maltose both compared with ACSP50_WT are shown. In ACSP50_Δ*malT*, no *malT* gene is present, whereas ACSP50_OE*malT* contains two copies of *malT*, one in the genome and one on the integrated plasmid. Heatmap of the fold changes of transcript abundance for the genes in the genomic region surrounding *malT* was derived from whole-genome microarray (Mal vs. Glc) and reverse transcription quantitative PCR (RT-qPCR) data (ACSP50_Δ*malT* (Mal) and ACSP50_OE*malT* (Mal)). Green color indicates increased transcription (for “Mal vs. Glc”: green = increased on maltose). Significance value of *p* < 0.05 is marked with a single asterisk, *p* < 0.01 with two asterisks, and *p* < 0.001 with three asterisks (*t* test, two-sample, Holm). The results of the RT-qPCR are listed in Supplemental Table S7

The results of the whole-genome microarrays were confirmed with RT-qPCR for the genes of the MRLGR, since RT-qPCR is more sensitive compared with the microarray technique. The genes, which were found to be less transcribed in the microarray data (Fig. 5), were confirmed to be downregulated by RT-qPCR data (Fig. 6). Strikingly, for 10 further genes, a significantly decreased transcription was determined. Thereby, all genes, which were previously described as co-regulated as well as upregulated on maltose, could be identified to be significantly less transcribed in ACSP50_Δ*malT* using RT-qPCR. Only *ACSP50_3948* (“hypothetical protein”) was found to be co-regulated along with the other genes of the MRLGR, but not influenced by the deletion of *malT*. In conclusion, 42 of 51 genes inside the MRLGR were found to be significantly less transcribed in the deletion mutant ACSP50_Δ*malT*.

In addition, the transcript levels of the MRLGR genes were measured for *malT* overexpression (ACSP50_OE*malT*) using the strong promoter P_*gapDH*_ from *Eggerthella lenta* (Schaffert et al. 2019a) compared with the empty vector control (Fig. 6 and Supplemental Table S7).

In total, 39 of the 51 genes were identified to be significantly upregulated in the *malT* overexpression strain ACSP50_OE*malT*. All of these 39 genes were also identified to be downregulated in ACSP50_Δ*malT*, except for *ACSP50_3906* and *ACSP50_3926* (Fig. 6), which are downregulated in ACSP50_Δ*malT* but show no significant transcriptional changes through *malT* overexpression (Fig. 6).

Looking at all datasets, the results match the proposed operon structure of the MRLGR. Genes transcribed in the same operon show the same transcription trends under the different tested conditions. The genes, which are transcribed in the same operon together with *malT* (*ACSP50_3912* to *ACSP50_3915*), show 10.000-fold to 3.000-fold decreased transcription compared with the wild type strain both cultivated on maltose minimal medium. In contrast to that, an overexpression of *malT* leads to an increased transcription of all genes in the operon (fold changes 2.53 to 6.92). The genes of the operon *ACSP50_3919* to *ACSP50_3924* are significantly “downregulated” in ACSP50_Δ*malT* compared with the wild type strain (fold changes 0.001 to 0.011) on maltose minimal medium, whereas an overexpression of *malT* (ACSP50_OE*malT*) leads to an increased transcription of this operon of 5.02 to 10.22 fold.

The same effect was found for operons *ACSP50_3900* to *ACSP50_3903*, *ACSP50_3908* to *ACSP50_3911*, *ACSP50_3929* to *ACSP50_3930*, *ACSP50_3936* to *ACSP50_3937*, *ACSP50_3938* to *ACSP50_3940*, and *ACSP50_3949* to *ACSP50_3950*. In ACSP50_Δ*malT*, a decreased transcription was observed, whereas an overexpression of *malT* leads to an increased transcription of these operons.

The operon *ACSP50_3944* to *ACSP50_3947* shows only partly this effect. Since deletion of *malT* leads to a decreased transcription of all genes in the operon, an overexpression does not affect transcription of the last gene inside the operon *ACSP50_3944*. However, since this gene is the last gene in this operon, this effect could be explained by less transcription of operon’s last genes due to shortened transcripts.

Interestingly, most of the genes which were not observed to be transcribed coordinately with the transcriptional activator gene *malT* were found to be less influenced regarding their respective transcription in ACSP50_Δ*malT* and ACSP50_OE*malT* compared with ACSP50_WT. These genes are *ACSP50_3905*, *ACSP50_3925* to *ACSP50_3927*, *ACSP50_3941* to *ACSP50_3943*, and *ACSP50_3948*, which show mostly no or a less strong effect regarding the *malT* expression level (Supplemental Table S6). This trend also correlates with the respective transcription level on maltose compared with glucose. Genes in this genomic region which seem to be transcribed coordinately and affected by the *malT* expression level show an increased transcription on maltose compared with glucose, whereas the abovementioned genes do not show any difference in transcription on maltose compared with glucose. An overview about all transcriptomic studies regarding the MRLGR is given in Supplemental Table S6. In conclusion, 37 genes were identified to be influenced by the expression level of MalT.

## Discussion

A MRLGR was identified by expression dynamics analysis. A total of 41 of 51 genes inside this MRLGR were found to be transcribed coordinately, showing a continuously increasing transcription during growth (Droste et al. 2020). Therefore, it can be assumed that these genes are co-regulated on a transcriptional level. A conserved palindromic sequence motif (5′-TCATCC-5 nt-GGATGA-3′) overlapping the -35 region of the corresponding promoter was identified upstream of 17 TSS of the MRLGR genes. This sequence motif partly matches the binding motif of the transcriptional activator MalT in *E. coli* and *Klebsiella pneumoniae* described as a repeat of a 5′-GGA(T/G)GA core hexanucleotide, bordered by two G residues on both sides 5′ GGGGA(T/G)GAGG (Richet and Raibaud 1989; Vidal-Ingigliardi et al. 1991; Boos and Shuman 1998). In *E. coli*, the 5′ end of this so-called MalT box was identified at position -34.5 to -35.5 in relation to the TSS, which overlaps with the -35 region of the corresponding promoters (Boos and Shuman 1998). The distance to the TSS of the potential regulatory sequence identified in *Actinoplanes* sp. SE50/110 is in good accordance with that 34.4 ± 1.0 nt. MalT is the ATP-dependent transcriptional activator of the maltose regulon in *E. coli* (Richet and Raibaud 1989). MalT was found to be maltotriose-dependent in *E. coli*. The genes of the MRLGR seem to be transcribed dependent on carbon source, activated on maltose, and repressed on glucose. Protein similarity of MalT in *E. coli* and in *Actinoplanes* sp. SE50/110 suggests that ACSP50_3915 also contains a maltose- or maltotriose-binding domain, as it was described for *E. coli*.

MalT-like regulators are widespread over different bacteria (Supplemental Fig. S3). Strikingly, two MalT-like regulators (*ACSP50_3915* and *ACSP50_3917*) were found in the MRLGR in *Actinoplanes* sp. SE50/110, of which *ACSP50_3915* shows the highest similarity to the *malT* gene of *E. coli* (Supplemental Fig. S2). Except for these two proteins, no other homologs (compared with MalT in *E. coli*) were identified in the *Actinoplanes* sp. SE50/110 genome. Furthermore, for gene deletion of *ACSP50_3917* in *Actinoplanes* sp. SE50/110, only slight effects on genes of the MRLGR were found (unpublished data). This could be due to an indirect effect of this regulator on the MRLGR genes. Therefore, it can be assumed that *ACSP50_3915* is the main transcriptional activator of the MRLGR and binds to the identified motifs in the -35 promoter region of these genes. Interestingly both regulators seem to have no effect on the genes of the maltose metabolism.

This could be confirmed by different transcriptomic studies on ACSP50_WT as well as deletion and overexpression mutants of *malT*. The deletion of *malT* leads to a significantly decreased transcription of 42 of these 51 genes, whereas overexpression of *malT* leads to a significantly increased transcription of at least 39 genes on maltose minimal medium. This results in a number of at least 37 genes, which are regulated by MalT, since both deletion and overexpression lead to significantly different transcription levels of these genes. In general, genes which were not affected by MalT seem to be less conserved in the MRLGR, as these genes could not be identified in similar genomic regions in other bacteria (Fig. 3 and Supplemental Table S4) and they were found to be not co-regulated or increased transcribed on maltose compared with glucose (Supplemental Table S6). This confirms the maltose dependency of the MalT regulon.

Interestingly, the MRLGR contains no genes encoding enzymes or proteins involved in maltose metabolism. Furthermore, none of the genes described for maltose utilization or transport (Schaffert et al. 2019a, 2019b) was found to be affected by deletion or overexpression of *malT* in *Actinoplanes* sp. SE50/110, and no similar sequence to the described MalT binding site was found upstream of these genes (data not shown).

Therefore, it can be assumed that transcription of genes involved in the maltose utilization is regulated MalT independently, although transcription of *malT* itself and therefore the genomic region *ACSP50_3900* to *ACSP50_3950* shows increased transcription on maltose (Fig. 6; Schaffert et al. 2019a, 2019b). Previous studies showed that transcription of the supposed maltose importer MalEFG is not regulated by a MalR homolog as it is described in *E. coli* (Wolf et al. 2017a). Regulation of maltose metabolism seems to be different in *Actinoplanes* sp. SE50/110 compared with other bacteria like *E. coli*. Therefore, the expression of several genes involved in maltose metabolism seems to be constitutive, since transcripts and proteins could be identified independently on supplied carbon source (Schaffert et al. 2019a, 2019b; Wolf et al. 2017a; Wendler et al. 2016). In contrast to that, the expression of the MRLGR, which in turn seems to be not involved in carbon metabolism, is highly influenced by the presence of maltose. Since maltose metabolism is closely connected to acarbose biosynthesis (Schaffert et al. 2019a, 2019b; Wendler et al. 2016), it could be assumed that gene products of the MRLGR are involved in the biosynthesis of acarbose precursors or related pathways.

However, the annotated function of most of the genes inside of this genomic region is unclear. Interestingly, several genes located in this genomic region were also found to be located in close proximity to each other in other organisms, like *P. ferrugineum*, *C. caeruleus*, and *K. cinnamomea*. Indeed, the arrangement and order of the homologous genes in these soil bacteria differ from *Actinoplanes* sp. SE50/110. Furthermore, several genes present in the MRLGR are not present in the other analyzed genomes, not even in close relatives like *A. missouriensis*. However, it was shown that the majority of these non-conserved genes are not regulated by MalT. Since these genes are strictly regulated dependent on maltose, it can be assumed that the corresponding proteins are needed especially on maltose. Indeed, the analyses using BLAST revealed protein functions for 10 gene products connected to the amino acid metabolism, such as arginine biosynthesis. Nevertheless, the presence of several homologs in the *Actinoplanes* sp. SE50/110 genome suggests that these genes are not mainly responsible for this biosynthetic pathway. A maltose-dependent regulation of these amino acid biosynthesis genes could not be explained. No common metabolic pathway could be identified for the annotated function of the conserved gene products of this genomic region. Most of the encoded proteins are enzymes or transport-related proteins involved in amino acid biosynthesis. However, also several genes annotated as hypothetical or uncharacterized proteins were reported. Since all strains containing parts of this genomic region were found in similar habitats, it can be assumed that this genomic region is involved in the metabolism of substrate specific for their respective soil habitat. As a soil bacterium, isolated from coffee plantation in Kenia (Frommer et al. 1975), a special nutrient supply of *Actinoplanes* sp. SE50/110 could be a reason for a sugar-dependent regulation of genes involved in amino acid uptake, peptide degradation, and amino acid biosynthesis. A close connection of sugar and amino acid metabolism has been reported for prokaryotes (Gänzle et al. 2007), as well as eukaryotes (Binder 2010; Rennie and Tipton 2000). In plants, several regulatory effects of sugar on specific parts of the amino acid metabolisms have been shown (Pratelli and Pilot 2014). A further explanation could be that gene products of the MRLGR are involved in biosynthesis of a metabolite, which is not essential. Therefore, it could be regulated dependent on availability of maltose as an indicator of good environmental conditions. However, the analysis of the MRLGR for secondary metabolite genes using the web tool antiSMASH 5.0 (Blin et al. 2019) revealed no hits (data not shown).

In conclusion, it can be assumed that this genomic region harbors genes important for specific habitats of *Actinoplanes* sp. SE50/110. As it can be found partly in other soil bacteria, which occur in similar environments, the proteins encoded in this genomic region could be involved in uptake and degradation of specific nutrients or in production of an optional metabolite.

## Electronic supplementary material

ESM 1 (XLSX 1919 kb)

ESM 2 (PDF 448 kb)

## Data Availability

The microarray datasets generated in this study can be found in the ArrayExpress database (www.ebi.ac.uk/arrayexpress) under accession E-MTAB-8815. All other data supporting the conclusion of this study are included in the article and its additional files.
