# Supplementary material for: A maltose-regulated large genomic region is activated by the transcriptional regulator MalT in Actinoplanes sp. SE50/110
Source: Appl Microbiol Biotechnol. 2020 Sep 28;104(21):9283–94. doi: 10.1007/s00253-020-10923-2 (PMC7567727; doi:10.1007/s00253-020-10923-2)
Supplement: Supplementary file 2 — (PDF 448 kb) [file 253_2020_10923_MOESM2_ESM.pdf]

Applied Microbiology and Biotechnology

Supplementary Figures

A maltose-regulated large genomic region is activated by the transcriptional regulator MalT in *Actinoplanes* sp. SE50/110

Julian Droste[1], Martin Kulisch[1], Timo Wolf[1], Lena Schaffert[1], Susanne Schneiker-Bekel[2], Alfred Pühler[2], and Jörn Kalinowski[1]§

Affiliations

1 Microbial Genomics and Biotechnology,

2 Senior Research Group in Genome Research of Industrial Microorganisms,

Center for Biotechnology, Bielefeld University, Universitätsstraße 27, 33615 Bielefeld, Germany

§ Corresponding author: Prof. Dr. Jörn Kalinowski (joern@CeBiTec.Uni-Bielefeld.DE)

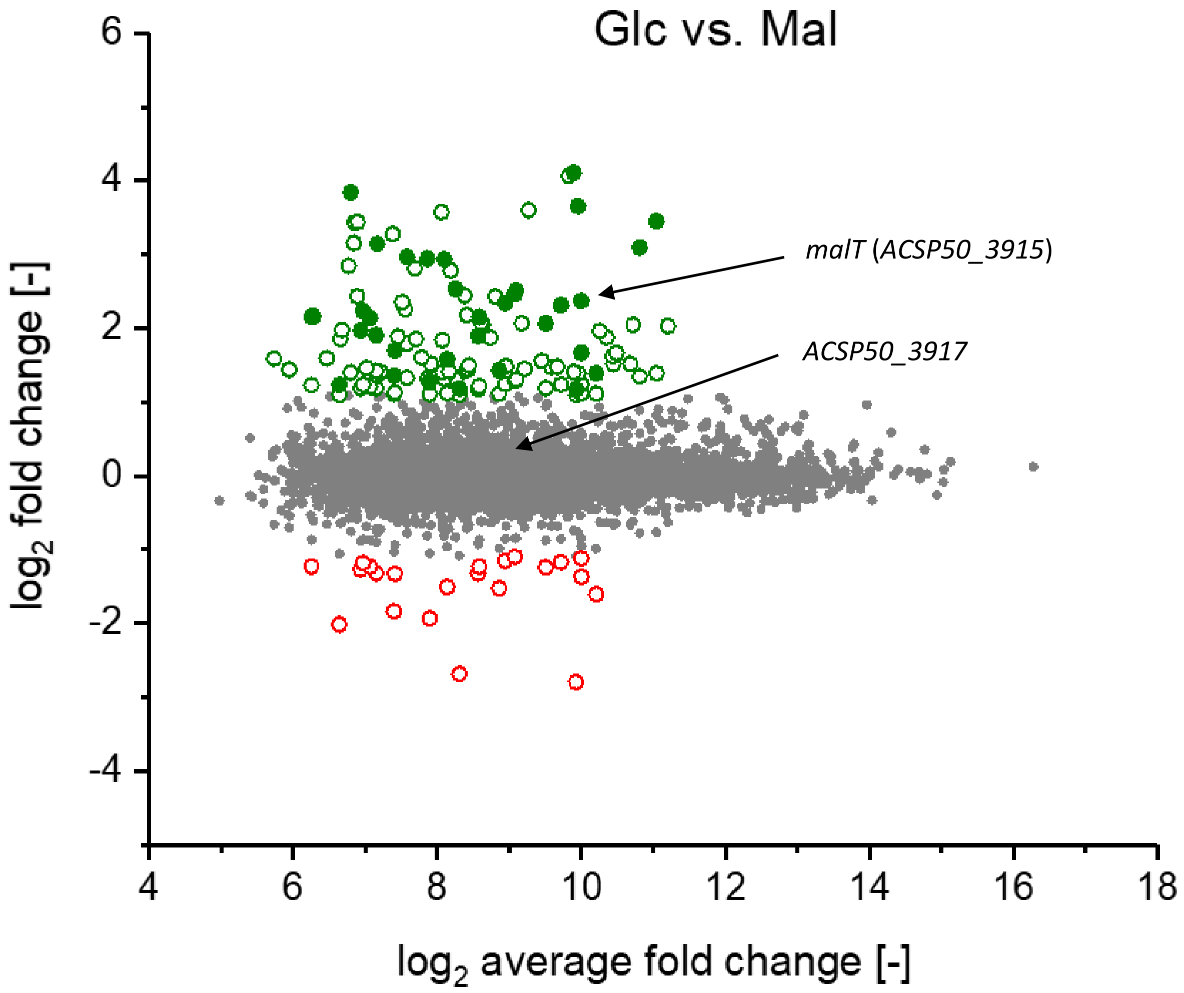

Supplementary Figure S1: Differential transcriptome analysis of *Actinoplanes* sp. SE50/110 (ACSP50_WT) grown on maltose compared to glucose as a carbon source. Samples were taken from shake flask cultivation in minimal medium supplemented with maltose and glucose respectively. Sampling point was the middle of the growth phase (after 72 h). For transcriptome analysis RNA of three biological replicates was used. Ratio/intensity plot from whole genome microarrays. Green and red dots represent genes with significantly different transcript levels in ACSP50_WT grown on maltose compared to glucose (*M*-value >1.1 or < -1.1 respectively; p_{adj}-value >0.05). Filled dots show genes of the MRLGR region.

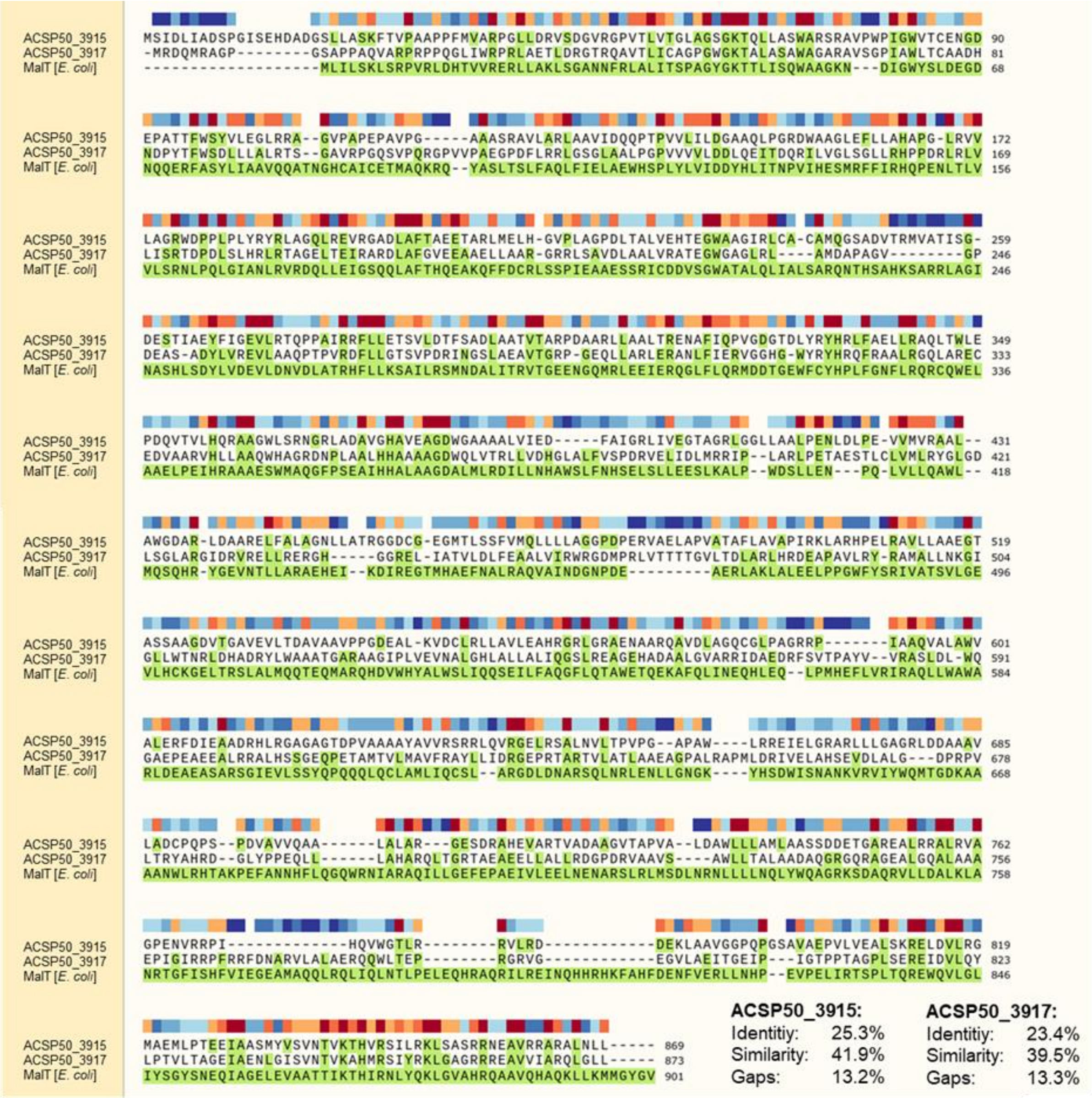

Supplementary Figure S2: Sequence alignment of the MalT-like regulator proteins (ACSP50_3915 and ACSP50_3917) encoded by the MRLGR in *Actinoplanes* sp. SE50/110 compared to the MalT protein of *Escherichia coli* (NCBI accession: NP_417877) using MUSCLE algorithm. Identical amino acids are colored in green. Consensus amino acids of all three proteins are color-coded above. Summary about similarity, identity and gaps are given at the bottom.

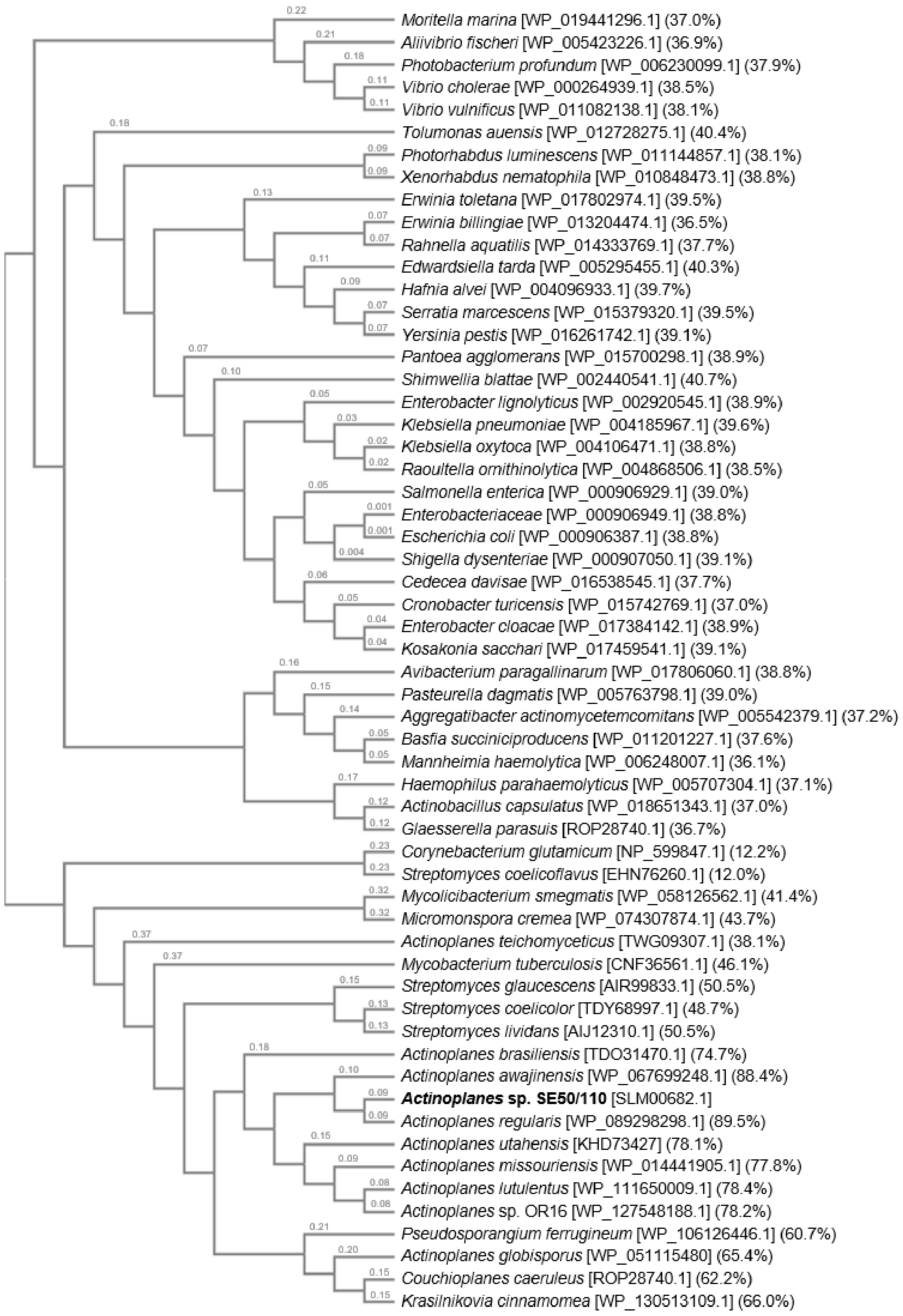

Supplementary Figure S3: Comparison of the MalT proteins in different bacteria. The accession number of the respective protein is indicated in square brackets. The similarity compared to ACSP50_3915 determined with BLASTP is given in round brackets.
